# Cytotoxic Activity of Aplykurodin A Isolated From *Aplysia kurodai* against *AXIN1*-Mutated Hepatocellular Carcinoma Cells by Promoting Oncogenic β-Catenin Degradation

**DOI:** 10.3390/md18040210

**Published:** 2020-04-13

**Authors:** Jaehoo Lee, Wei Zhou, MinKyun Na, Sangtaek Oh

**Affiliations:** 1 Department of Bio and Fermentation Convergence Technology, BK21 PLUS Program, Kookmin University, Seoul 136-702, Korea; wogn1208@gmail.com; 2 College of Pharmacy, Yanbian University, Yanji 133002, China; zhouwei8452@163.com; 3 College of Pharmacy, Chungnam National University, Daejeon 34134, Korea

**Keywords:** hepatocellular carcinoma (HCC), aplykurodin A, Wnt/β-catenin signaling, apoptosis, autophagy

## Abstract

Dysregulation of the Wnt/β-catenin signaling pathway is involved in the development of human hepatocellular carcinoma and has thus emerged as a therapeutic target for this malignant tumor. In this study, we employed sensitive cell-based assays to identify aplykurodin A isolated from *Aplysia kurodai* as an antagonist of Wnt/β-catenin signaling. Aplykurodin A inhibited β-catenin responsive transcription, which was stimulated by a Wnt3a-conditioned medium or a glycogen synthase kinase 3β inhibitor by accelerating intracellular β-catenin degradation. Aplykurodin A downregulated the level of oncogenic β-catenin and decreased the expression of β-catenin-dependent gene, leading to inhibition of human hepatoma Hep3B and SNU475 cell proliferation. Moreover, apoptosis and autophagy were elicited by aplykurodin A, as indicated by an increase the number of Annexin V-FITC-stained cells and the formation of microtubule-associated protein 1 light chain 3 puncta, respectively, in Hep3B and SNU475 cells. Our findings suggest that aplykurodin A provides a novel therapeutic strategy for human hepatocellular carcinoma via stimulation of oncogenic β-catenin degradation.

## 1. Introduction

Hepatocellular carcinoma (HCC) is the most common type of liver cancer and the third leading cause of cancer-mediated mortality worldwide [1]. Recent therapies for this malignancy rely on surgical resection, by which only early stage HCC patients can be cured [2]; however, current diagnosis for HCC often fails to sense the early stage HCC [3]. In addition, sorafenib, an inhibitor of receptor tyrosine kinase, has been used for the systemic treatment of advanced HCC, but most of patients do not exhibit the desired response to this therapeutics [4]. Therefore, it is still necessary to develop new therapeutic strategies that are based on defined molecular lesions.

β-Catenin is an essential constituent of the Wnt/β-catenin pathway, which regulates cell growth, differentiation and development [5,6,7] and its turnover is tightly controlled by ubiquitin-dependent proteolysis. Normally, casein kinase 1 (CK1) and glycogen synthase kinase 3β (GSK-3β) phosphorylate the amino-terminal region of β-catenin in a complex with adenomatous polyposis coli (APC) and Axin [8,9]. Then, phosphorylated β-catenin, which is recognized F-box β-transducin repeat-containing protein (β-TrCP) E3 ubiquitin ligase, is ubiquitinated and degraded by proteasome [10,11]. 

The aberrant β-catenin accumulation, which is caused by mutation in *AXIN* gene or infection of hepatitis B virus, is frequently observed in hepatocellular carcinoma [12,13]. Then, β-catenin moves to the nucleus and binds to T-cell factor/lymphocyte enhancer factor (TCF/LEF) family transcription factor, leading to activation of β-catenin-dependent genes including cyclin D1, c-myc, and metalloproteinase-7 (MMP-7), which are involved in tumorigenesis and metastasis [14,15,16,17]. Hence, degradation of oncogenic β-catenin may be a plausible strategy for treating hepatocellular carcinoma. 

The sea hare, *Aplysia kurodai* (family Aplysiidae), is distributed in the coasts of Northeast Asia. Because of its unique texture and flavor, *A. kurodai* has been consumed as seafood in South Korea. The sea hare is also used as a traditional medicine to treat inflammation and wounds. Previous study revealed that *Aplysia* species protect themselves by releasing toxic compounds stored in the digestive glands [18], and such toxic metabolites could have high potential in developing anti-tumor agents. Until now, tens of chemical constituents have been reported from the sea hare, of which aplysin and a benzopyrrole could inhibit the proliferation and induce apoptosis in human gastric cancer cells [19]. The macrolides, aplyronines A-C, isolated from *A. kurodai* were reported to have cytotoxic activity against human cervical cancer cells [20]. Moreover, halogenated sesquiterpenes such as laurinterol, laurinterol acetate and debromolaurinterol found in the species showed cytotoxic activity against HeLa cells [21]. Aplykurodin A, a degraded sterol originally discovered from *A. kurodai* in 1986 [22], was obtained as a major secondary metabolite (yield 0.037%) in our large-scale chemical investigation on the species. Despite various biological activities, in particular cytotoxicity, of *A. kurodai*-derived compounds, there are few reports on its biological activity on aplykurodin A. Based on the fact that a degraded sterol, 3-*epi*-aplykurodinone B, displayed cytotoxicity against human tumor carcinoma cells, our study was focused on the evaluation of cytotoxic activity against HCC in association with Wnt/β-catenin signaling pathway. In the present study, we demonstrated that aplykurodin A antagonized Wnt/β-catenin signaling and inhibited proliferation of hepatocellular carcinoma cells by destabilizing intracellular β-catenin.

## 2. Results

### 2.1. Aplykurodin A Suppresses the Wnt/β-Catenin Pathway

**Figure 1:**
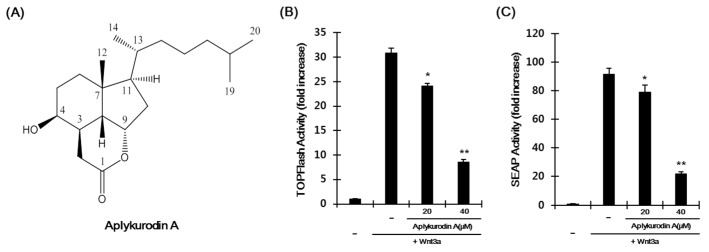
Recognizing aplykurodin A as a repressor of Wnt/β-catenin signaling. (**A**) The structure of aplykurodin A. (**B**,**C**) Inhibition of β-catenin responsive transcription (CRT) by aplykurodin A. After incubation of HEK293 reporter cells with either DMSO or aplykurodin A (20 and 40 μM) in the presence of Wnt3a-CM for 15 h, firefly luciferase (FL) (**B**) and HEK293-secreted alkaline phosphatase (SEAP) (**C**) activities were determined. These results represent the mean ± S.D. of three independent experiments. * *p* < 0.05 and ** *p* < 0.01, compared with the Wnt3a-CM-treated control group.

To examine whether aplykurodin A inhibits the Wnt/β-catenin pathway, we used HEK293-firefly luciferase (FL) reporter cells previously established [23]. Incubation of HEK293-FL reporter cells with Wnt3a-conditioned medium (Wnt3a-CM) increased FL activity and treatment with aplykurodin A produced a concentration-dependent decrease in β-catenin responsive transcription (CRT) without detectable cytotoxicity (Figure 1A,B and Figure S1). We confirmed the inhibitory effect of aplykurodin A on CRT using HEK293-secreted alkaline phosphatase (SEAP) reporter cells previously established [24]. As expected, aplykurodin A reduced SEAP activity induced by Wnt3a-CM in a dose-dependent manner (Figure 1C). In contrast, p53 and NF-κB reporter activities were largely unaffected by aplykurodin A (Figure S2A,B). These results indicate that aplykurodin A is a specific antagonist of Wnt/β-catenin signaling.

### 2.2. Aplykurodin A Promotes Proteasomal Degradation of β-Catenin

**Figure 2:**
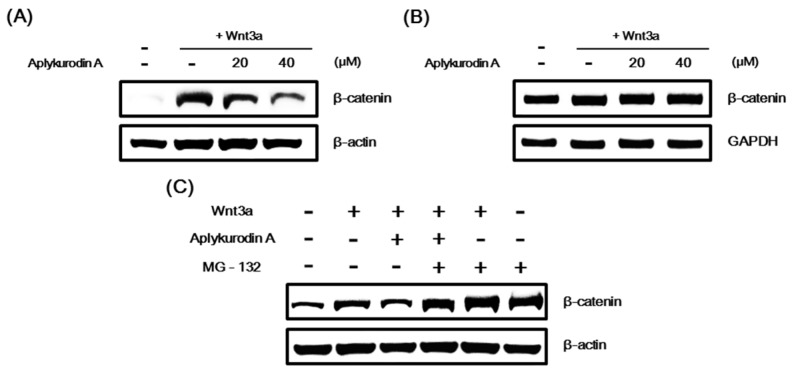
Aplykurodin A promotes proteasomal β-catenin degradation. (**A**) After treatment of HEK293-FL cells with either DMSO or aplykurodin A (20 and 40 μM) in the presence of Wnt3a-CM for 15 h, cytosolic proteins were analyzed by Western blotting with anti-β-catenin antibody. (**B**) After treatment of HEK293-FL cells with either DMSO or aplykurodin A (20 and 40 μM) in the presence of Wnt3a-CM for 15 h, semi-quantitative RT-PCRs for β-catenin and glyceraldehyde 3-phosphate dehydrogenase (GAPDH) were carried out with total RNA. (**C**) HEK293-FL reporter cells were treated with either DMSO or aplykurodin A (20 μM) and then exposed to MG-132 (10 μM) for 8 h. Cytosolic proteins were analyzed by Western blotting with anti-β-catenin antibody. (**A**,**C**) the blots were re-probed with anti-actin antibody. The results are representative of three independent experiments.

In Wnt/β-catenin signaling, CRT primarily relies on the amount of intracellular β-catenin [25] that is controlled by a proteasomal degradation [10]. Since aplykurodin A suppressed Wnt3a-induced CRT, we tested whether aplykurodin A modulated the β-catenin protein level. Western blot analysis showed that aplykurodin A decreased the level of intracellular β-catenin, which was activated by Wnt3a-CM, in HEK293-FL reporter cells (Figure 2A). Under these conditions, aplykurodin A did not affect β-catenin mRNA level (Figure 2B). We next tested whether the proteasome was involved in β-catenin downregulation induced by aplykurodin A. As depicted in Figure 2C, the amount of intracellular β-catenin was consistently reduced by aplykurodin A. However, this β-catenin downregulation was abrogated in the presence of MG-132, a proteasome inhibitor. Taken together, these findings indicate that aplykurodin A antagonized the Wnt/β-catenin pathway through promotion of proteasome-dependent β-catenin degradation without affecting β-catenin gene expression. 

### 2.3. Aplykurodin A Promotes β-Catenin Degradation Through a Mechanism Independent of GSK-3β

**Figure 3:**
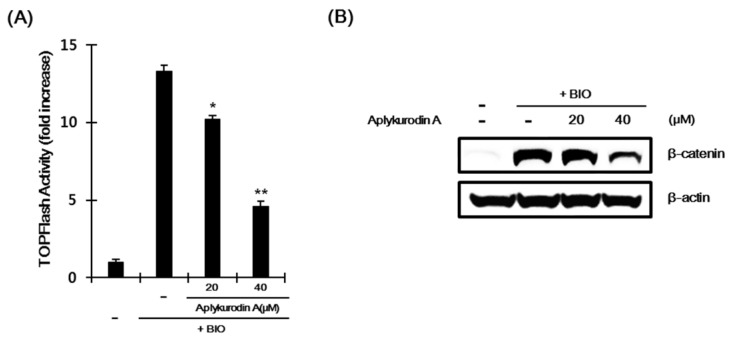
GSK-3β is not required for aplykurodin A-promoted β-catenin degradation. (**A**) After incubation of HEK293 reporter cells with either DMSO or aplykurodin A (20 and 40 μM) in the presence of 0.75 μM of 6-bromoindirubin-3′-oxime (BIO) for 15 h, FL activity was measured. The results represent the mean ± S.D. of three independent experiments. * *p* < 0.05 and ** *p* < 0.01, compared with the BIO-treated control group. (**B**) After incubation of HEK293 reporter cells with either DMSO or aplykurodin A (20 and 40 μM) in the presence of 0.75 μM of BIO for 15 h, cytosolic proteins were analyzed by Western blotting with anti-β-catenin and anti-β-actin antibodies. The results are representative of three independent experiments.

GSK-3β catalyzes β-catenin phosphorylation at Ser33, Ser37, and Thr41 residues, which is prerequisite event for acceleration of β-catenin turnover. We, thus, investigated whether GSK-3β is involved in aplykurodin A-induced β-catenin degradation. As previously reported [26], incubation of HEK293-FL reporter cells with 6-bromoindirubin-3′-oxime (BIO), a GSK-3β inhibitor, led to an increase in CRT. Under this condition, aplykurodin A still suppressed CRT (Figure 3A). Western blot analysis revealed that aplykurodin A reduced the intracellular β-catenin level induced by BIO in HEK293-FL reporter cells (Figure 3B). These findings suggest that GSK-3β is not required for aplykurodin A-induced β-catenin degradation. In addition, we found that the level of mutant β-catenin lacking N-terminal region (ΔN β-catenin) was not altered in HepG2 HCC cell by aplykurodin A treatment (Figure S3). Taken together, these results indicate that N-terminal phosphorylation, which is not catalyzed by GSK-3β, is still necessary for aplykurodin A-mediated β-catenin degradation. 

### 2.4. Aplykurodin A Has Anti-Porliferative Effects in AXIN1-Mutated HCC Cells

**Figure 4:**
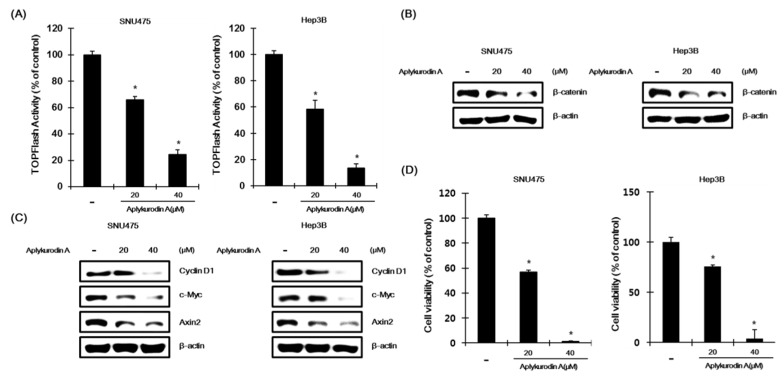
The effects of aplykurodin A in SNU475 and Hep3B cells. (**A**) After incubation of TOPFlash and pCMV-*Renilla* luciferase (RL) plasmids co-transfected SNU475 and Hep3B cell with either DMSO or aplykurodin A (20 and 40 μM), FL and RL activities were determined. TOPFlash activity is normalized to RL activity. The results represent the mean ± S.D. of three independent experiments. * *p* < 0.01 compared with the DMSO control group. (**B**,**C**) After incubation of SNU475 and Hep3B cells with either DMSO or aplykurodin A (20 and 40 μM), cytosolic proteins were analyzed by Western blotting with anti-β-catenin and anti-β-actin antibodies (**B**) and cell extracts were immunoblotted with anti-cyclin D1, anti-c-myc, anti-Axin2 antibodies, and anti-β-actin (loading control) antibodies (**C**). The results are representative of three independent experiments. (**D**) The effect of aplykurdin A on cell viabilities of SNU475 and Hep3B cells. Details are described in Materials and Methods. The results represent the mean ± S.D. of three independent experiments. * *p* < 0.01 compared with the DMSO control group.

Because abnormal CRT activation is often observed in human hepatocellular carcinoma (HCC), we evaluated whether aplykurodin A was able to downregulate CRT in SNU475 and Hep3B hepatoma cells that displayed elevated CRT because of an inactivation mutation in *AXIN1*, a critical component of destruction complex [27,28]. When SNU475 and Hep3B cells transfected with TOPFlash were incubated with aplykurodin A, we observed that CRT was decreased by aplykurodin A (Figure 4A). In addition, Western blot analysis showed that the level of intracellular β-catenin was downregulated in response to aplykurodin A in SNU475 and Hep3B cells (Figure 4B). Next, to investigate whether aplykurodin A affects β-catenin dependent gene expression, SNU475 and Hep3B cells, which has a mutation in the *AXIN1* gene, were incubated with aplykurodin A. Western blot analysis revealed that aplykurodin A downregulaed the expression of β-catenin-dependent genes, cyclin D1, c-myc, and axin-2 (Figure 4C). Given that aplykurodin A promoted oncogenic β-catenin degradation, we tested its effect on the growth of *AXIN1*-mutated hepatoma cells. SNU475 and Hep3B cells. As expected, aplykurodin A efficiently decreased cell viabilities of SNU475 and Hep3B cells in a concentration-dependent manner (Figure 4D). Under these conditions, the growth of IMR90 and WI38 cells, normal fibroblasts, were largely unaffected by aplykurodin A (Supplement Figure S4A,B). 

### 2.5. Aplykurodin A Induces Apoptosis in SNU475 and Hep3B Cells

**Figure 5:**
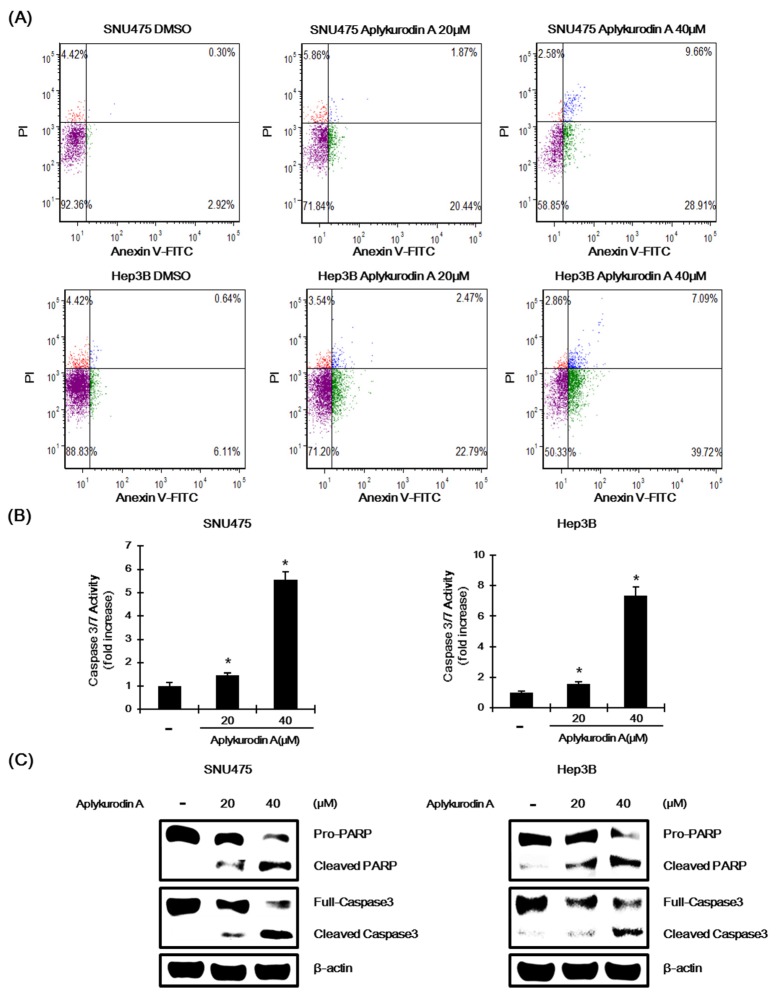
Aplykurodin A induces apoptosis in SNU475 and Hep3B cells. (**A**) After incubation of SNU475 and Hep3B cells with either DMSO or aplykurodin A (20 and 40 μM) for 48 h, Annexin V-FITC and propidium iodide (PI) stained cells were analyzed by a Cellometer cytometer. (**B**) After incubation of SNU475 and Hep3B cells with either DMSO or aplykurodin A (20 and 40 μM) for 48 h, caspase3/7 activity was determined. Details were described in Materials and Methods. The results represent the mean ± S.D. of three independent experiments. * *p* < 0.01 compared with the DMSO control group. (**C**) After incubation of SNU475 and Hep3B cells with either DMSO or aplykurodin A (20 and 40 μM) for 48 h, cell extracts were immunoblotted with anti-caspase-3, anti-poly (ADP-ribose) polymerase (PARP) and anti-actin (loading control) antibodies. The results are representative of three independent experiments.

To reveal underlying mechanism of aplykurdoin A-mediated growth inhibition, we examined whether aplykurodin A induces apoptosis in *AXIN1*-mutated hepatoma cells. SNU475 and Hep3B cells were exposed to aplykurodin A and then the number of apoptotic cells was counted using Annexin V/PI staining. The proportion of Annexin V positive or Annexin V/PI double positive cells was significantly increased in a concentration-dependent manner. Moreover, treatment of these AXIN1-mutated hepatoma cells with aplykurodin A activated caspase-3/7, which is consistent with an increase in apoptosis (Figure 5B). Finally, incubation of SNU475 and Hep3B cells with aplykurodin A induced the proteolytic cleavage of pro-caspase-3 and poly (ADP-ribose) polymerase (PARP), biochemical markers of apoptosis (Figure 5C). These findings suggest that apoptosis contributes to aplykurodin A-mediated inhibition of proliferation in AXIN1-mutated hepatoma cells.

### 2.6. Aplykurodin A Induces Autophagy in SNU475 and Hep3B Cells

**Figure 6:**
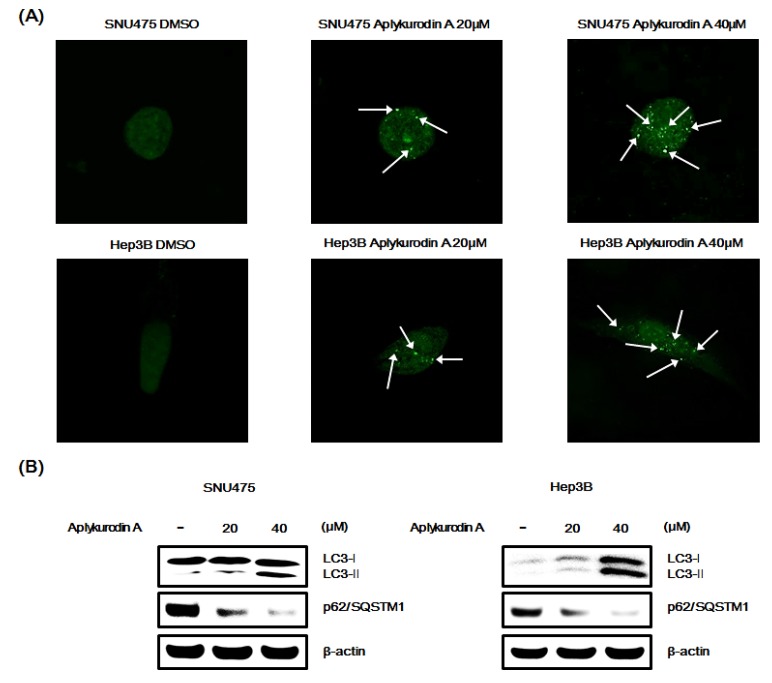
Aplykurodin A promotes autophagy in SNU475 and Hep3B cells. (**A**) After incubation of SNU475 and Hep3B cells with either DMSO or aplykurodin A (20 and 40 μM) for 48 h, the punctate distribution of GFP-LC3 were analyzed by confocal microscopy. (**B**) After incubation of SNU475 and Hep3B cells with either DMSO or aplykurodin A (20 and 40 μM) for 48 h, cell extracts were immunoblotted with anti-LC3, p62/SQSTM1, and anti-actin (loading control) antibodies. The results are representative of three independent experiments.

Several studies have reported that inhibition of β-catenin function promotes autophagy, a conserved catabolic pathway, in cancer cells [29,30]. Given that aplykurodin A decreased the amount of β-catenin protein, we postulated that aplykurodin A might stimulate autophagic cell death in AXIN1-mutated hepatoma cells. Thus, we determined formation of the autophagosome-associated light chain 3-II (LC3-II), an indication of autophagy, from the cytosolic microtubule-associated protein LC3-I in SNU475 and Hep3B hepatoma cells, ectopically expressed with a green fluorescence protein tag of LC3 (GFP-LC3). As depicted in Figure 6A, aplykurodin A induced redistribution of GFP-LC3 from a diffuse pattern to punctuate dots. Western blot confirmed that aplykurodin A promoted conversion of LC3-I to lipidated LC3-II and decreased the level of p62/SQSTM1, an LC3- interacting protein, in SNU475 and Hep3B cells (Figure 6B). These findings suggeste that autophagy is a possible mechanism by which aplykurodin A induces cell death in hepatoma cells.

## 3. Discussion

Because it is known that the sea hare *A. kurodai* has a chemical defense system that releases toxic metabolites, special attention is being paid to the anticancer potential of the *A. kurodai*-derived compounds. Aplykurodin A, isolated from *A. kurodai*, is a degraded sterol and has been obtained as a major secondary metabolite in our large-scale chemical investigation. Since one of the degraded sterols, 3-*epi*-aplykurodinone B, derived from *A. kurodai* was reported to have cytotoxicity against human cancer cells, our study was focused on the evaluation of its cytotoxic activity against HCC cells and the elucidation of its possible mechanism. In the current study, we demonstrated for the first time that aplykurodin A from *A. kurodai* induced oncogenic β-catenin degradation, thereby efficiently inhibiting growth of HCC cells. 

Axin1, a scaffolding protein, interacts with CK1, GSK-3β, APC, and β-catenin via separate domains [8] and coordinates the sequential phosphorylation of N-terminal motif of β-catenin, leading to ubiquintin-dependent proteasomal degradation of β-catenin [10]. In this study, several lines of evidences suggest that aplykurodin A-induced β-catenin turnover is distinct from above mechanism involving Axin1 and GSK-3β. We demonstrated that aplykurodin A downregulated intracellular β-catenin levels even in the presence of a GSK-3β inhibitor. Moreover, in SNU475 and Hep3B cells where the destruction-dependent pathway was impaired because of loss-of-function mutations in *AXIN1*, aplykurodin A was still able to stimulate degradation of β-catenin in SNU475 and Hep3B cells. 

Previous studies have demonstrated that protein kinase Cα (PKCα) and cyclin-dependent kinase 2 (CDK2)/cyclin A phosphorylate β-catenin at N-terminal residues (Ser33/37/Thr41) and N-terminal phosphorylation motif is essential for β-catenin degradation [31,32,33]. Likewise, we found that wild-type β-catenin was efficiently decreased in SNU475 and Hep3B cells by aplykurodin A whereas the amounts of mutant β-catenin lacking N-terminal region was largely unaffected in response to aplykurodin A, suggesting that N-terminal residues of β-catenin are required for aplykurodin A-mediated β-catenin degradation. Therefore, in addition to GSK-3β, other kinases, such as PKCα and CDK2 may be involved in proteasome-dependent β-catenin decomposition induced by aplykurodin A. 

Several studies have reported that tumor suppressor p53 involved in regulation of the Wnt/β-catenin pathway. Activation of p53 induces expression of Siah-1 E3 ubiquitin ligase, which promotes β-catenin degradation in a complex with APC. We reported previously that hexachlorophene accelerated β-catenin decomposition by activating the expression of Siah-1 in HCT116 and LS174T colon cancer cells [23]. In contrast, small molecules such as IWR-3 and XV939 stabilize Axin1 and increase the β-catenin decomposition, thereby inhibiting proliferation of DLD-1 colon cancer cells [34,35]. In this study, aplykurodin A was able to inhibit the growth of SNU475 and Hep3B hepatoma cells with both Axin and p53 mutations. 

The Wnt/β-catenin pathway regulates both autophagy and apoptosis [36,37]. Autophagy plays important roles in the cellular homeostasis mechanism against various cellular stresses through autophagosome and lysosome system [38]. Apoptosis, a conserved programmed cell death mechanism, is involved in the elimination of damaged or cancerous cells. Particularly, cytotoxic agents induce the intrinsic apoptotic pathway, which is characterized by activation of caspases-3/7 and PARP cleavage [39]. Autophagy and apoptosis may be cross-talked, either cooperatively or antagonistically, in response to a variety of anti-cancer therapeutics [40]. We found that the formation of LC3-II and the activation of caspase-3 were induced by aplykurodin A, thereby demonstrating that aplykurodin A promotes both autophagy- and apoptosis-mediated cell death in SNU475 and Hep3B hepatoma cells. 

In summary, we identified a novel Wnt/β-catenin signaling inhibitor aplykurodin A and demonstrated its anti-proliferative activity aganist HCC cells. Aplykurodin A promoted proteasomal β-catenin decomposition through a mechanism independent of GSK-3β and Axin1, major components of β-catenin destruction complex. In addition, aplykurodin A induced apoptosis and autophagy, thereby suppressing the growth of *AXIN1*-mutated HCC cells. Although aplykurodin A exhibited cytotoxic effect against HCC cells at high concentration, it is likely to be development into chemopreventive or therapeutic agent against CRT-positive cancers caused by *AXIN1* mutation.

## 4. Materials and Methods

### 4.1. Isolation of Aplykurodin A

*Aplysia kurodai* (35 kg, wet wt) were extracted by CH_2_Cl_2_/EtOH (1:1) three times under reflux. The extract (800 g) was suspended in water and partitioned successively with CH_2_Cl_2_, yielding CH_2_Cl_2_ (140 g) and water-soluble (620 g) fractions. The CH_2_Cl_2_ fraction (140 g) was subjected to silica gel vacuum liquid column chromatography (VLC) (1400 g) and eluted with stepwise gradient of *n*-hexane-EtOAc (30:1, 7:1, 4:1, 1.5:1), *n*-hexane-EtOAc-MeOH (1:1:0.2), CHCl_3_-MeOH (3:1), CHCl_3_-MeOH-H_2_O (1:1:0.1), and MeOH (100%) to give eight fractions (Fr. A-H). Fr. D (2.7 g) was combined with Fr. E (5 g) based on their similar TLC patterns and continue to separate into 10 fractions (Fr. DE-1—DE-10) by MPLC (C_18_ SNAP cartridge KP-C_18_-HS, 340 g) with a gradient of acetone-MeOH-H_2_O (17:17:66 → 47:47:6). Fraction DE-5 (1.02 g) was separated using silica gel column (1.5 cm × 80 cm) chromatography with a gradient of n-hexane-EtOAc-acetone (6.5:1:0.1 → 4:1:0.1) to give six subfractions (Fr. DE-5-1—DE-5-6). Fraction DE-5-6 (337.7 mg) was purified by a silica gel column (1.0 × 70 cm) and eluted with n-hexane-EtOAc-acetone (5:1:0.2) to get aplykurodin A (210 mg). Aplykurodin A was obtained as white amorphous powder. The protonated-molecular ion was detected at *m*/*z* 322.25 in ESI-MS, corresponding to the molecular formula C_20_H_34_O_3_. The ^1^H NMR spectroscopic data showed typical signals for two oxygenated methine protons at δ_H_ 5.06 (1H, d, *J* = 6.3 Hz, H-9), and 3.86 (1H, br s, H-4), one tertiary methyl group at δ_H_ 1.02 (3H, s, H-12), and three secondary methyl groups at δ_H_ 1.02 (3H, d, *J* = 6.4 Hz, H-14), 0.94 (6H, d, *J* = 6.6 Hz, H-19, H-20). The ^13^C NMR spectroscopic data displayed 20 carbon signals, which were assigned to one carboxyl group at δ_C_ 175.2 (C-1), two oxygenated methines at δ_C_ 82.5 (C-9) and 67.4 (C-4), one tertiary methyl at δ_C_ 23.4 (C-12), three secondary methyls at δ_C_ 23.2 (C-20), 22.9 (C-19), and 19.2 (C-14), in addition to one quaternary carbon, five methines, and seven methylenes. Based on the NMR spectroscopic data analysis (see the ^1^H and ^13^C NMR spectroscopic data assignment in Table S1), the compound was identified as aplykurodin A.

### 4.2. Cell Culture, Reporter Assay, Transfection, and Chemicals Used

HEK293, SNU475, and Hep3B cell lines (ATCC, Manassas, VA, USA) were cultured in the Dulbecco’s modified Eagle’s medium (DMEM) containing 10% fetal bovine serum (FBS) and 1% penicillin/streptomycin. The HEK293-FL, HEK293-SEAP reporter cells, and Wnt3a-CM were prepared as previously described [23,24]. The FL and SEAP assays were carried out using the dual luciferase assay kit (Promega, Madison, WI, USA) and the Phospha-Light assay kit (Applied Biosystems, Foster City, CA, USA), respectively, according to the manufacturer’s instructions. For reporter assay, HEK293-FL and HEK293-SEAP reporter cells were seeded into 96-well plate at density of 15,000 cells/well. Then, Wnt3a-CM and indicated concentrations of aplykurodin A, which was dissolved in DMSO at a concentration of 50 mM (stock solution), were added. After 15 h, FL and SEAP activities were measured by microplate reader (Victor V3, PerkinElmer, MA, USA). Transfections were conducted using Lipofectamine 2000 (Invitrogen, Carlsbad, CA, USA), according to the manufacturer’s instructions. MG-132 and BIO were obtained from Sigma-Aldrich (St. Louis, MO, USA). 

### 4.3. Western Blotting and Antibodies

Western blot analyses were performed as previously described [41]. Anti-β-catenin antibody was obtained from BD Transduction Laboratories (Palo Alto, CA, USA). Anti-axin2, anti-PARP, anti-caspase-3, anti-actin, and anti-lc3b antibodies were purchased from Cell Signaling Technology (Danvers, MA, USA). Anti-cyclin D1, anti-c-myc, and anti-p62 antibodies were obtained Santa Cruz Biotechnology (Dallas, TX, USA). 

### 4.4. RNA Extraction and Semi-Quantitative RT-PCR

RNA was isolated from HEK293-FL reporter cells using TRIzol Reagent (Invitrogen, Carlsbad, CA, USA), according to the manufacturer’s instructions. Complementary DNA (cDNA) synthesis, amplification, and polymerase chain reaction (PCR) were performed as previously described [32]. Amplified cDNA was electrophoresed on a 2% (*w*/*v*) agarose, stained with Dyne LoadingSTAR and detected using Gel document system (InGenius, Gel doc, CT, USA).

### 4.5. Cell Viability Assay

Cells were innoculated into 96-well plates and incubated with aplykurodin A (20 and 40 μM) for 48 h. The cell viability of each treated sample was measured in triplicate using the CellTiter-Glo assay kit (Promega) according to the manufacturer’s instructions. The value of time 0 was subtracted to calculate growth inhibition of hepatoma cells.

### 4.6. Apoptosis Analysis and Caspase-3/7 Assay

After treatment with aplykurodin A for 48 h, the cells were stained with ApoScanTM annexin V-FITC apoptosis detection kit (Promega), according to the instructions and then analyzed by Cellometer Vision image cytometer (Nexcelom Bioscience). The activity of caspase was determined using the Caspase-Glo 3/7 Assay (Promega) according to the manufacturer’s instructions.

### 4.7. Fluorescence Microscope Analysis of GFP-LC3

The GFP-LC3-transfected cells were incubated with aplykurodin A for 48 h and fixed with 4% paraformaldehyde in PBS at room temperature. Fluorescence signals were visualized and captured using the Leica 4000 confocal microscope.

### 4.8. Statistical Analysis

The Student’s t-test was used to compare the means between control and experimental groups. All experiments were performed three times. Statistical significance was set at *p* < 0.05 or *p* < 0.01. Results are presented as mean ± standard deviation (SD).

## Supplementary Materials

The following are available online at https://www.mdpi.com/1660-3397/18/4/210/s1, Figure S1: Aplykurodin A is not cytotoxic to HEK293 reporter cell., Figure S2: Aplykurodin A does not affect p53 and NF-B pathways, Figure S3: Aplykurodin A promotes β-catenin decomposition in HepG2 HCC cells with a mutant β-catenin lacking the N-terminal phosphorylation motif, Figure S4: Aplykurodin A is not cytotoxic to IMR90 and WI38 cells, Table S1: NMR Spectroscopic Data of aplykurodin A (300 MHz, methanol-*d*_4_).
