# Supplementary material for: Cytotoxic Activity of Aplykurodin A Isolated From Aplysia kurodai against AXIN1-Mutated Hepatocellular Carcinoma Cells by Promoting Oncogenic β-Catenin Degradation
Source: Mar Drugs. 2020 Apr 13;18(4):210. doi: 10.3390/md18040210 (PMC7230895; doi:10.3390/md18040210)
Supplement: Supplementary file 1 [file marinedrugs-18-00210-s001.pdf]

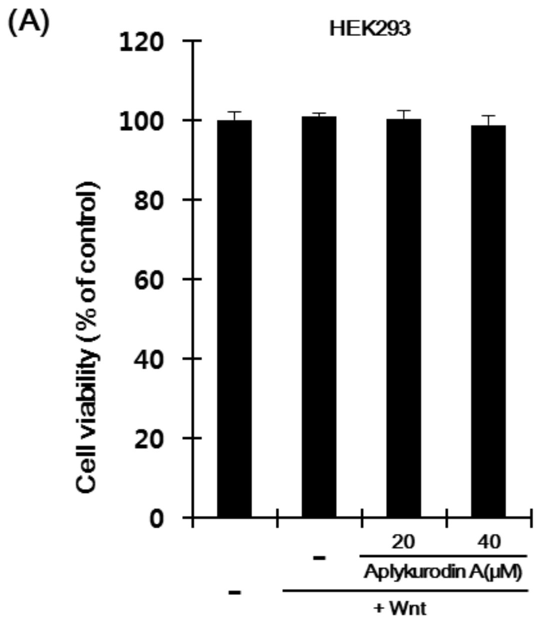

Figure S1. Aplykurodin A is not cytotoxic to HEK293 reporter cells. (A) HEK293 reporter cells were incub ated with aplykurodin A (20μM and 40μM) for 15 hours in the absence or presence of Wnt3a-CM, and cel l viability was measured by Cell titer-Glo.

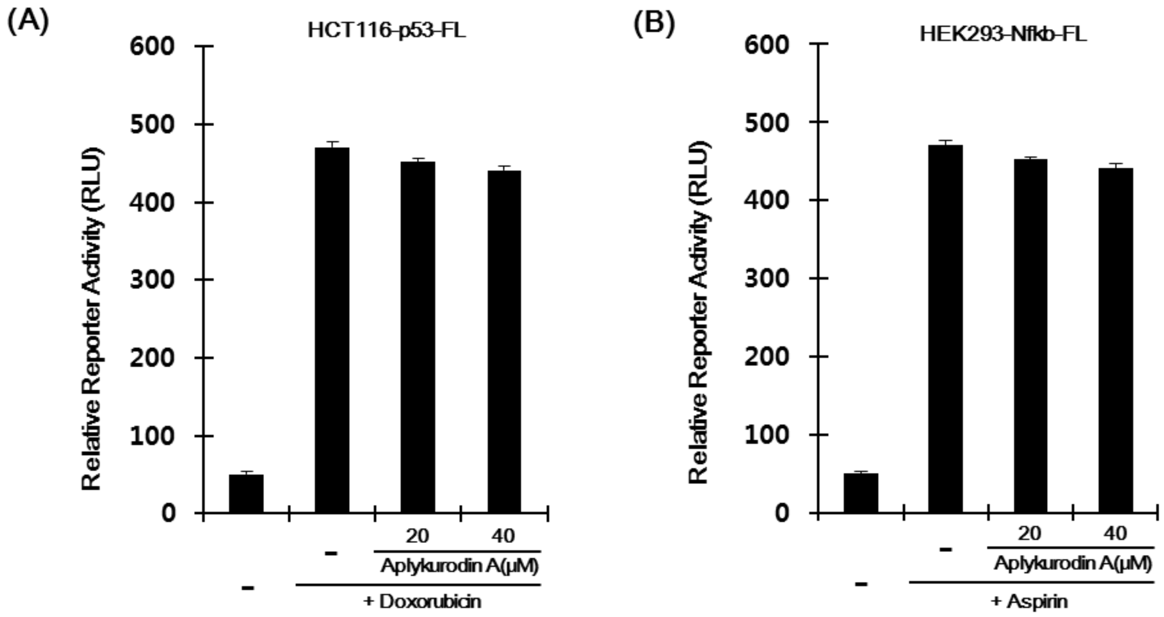

Figure S2. Aplykurodin A does not affect p53 and NF-B pathways. (A) HCT116 cells were co-transfected wi th p53-FL and pCMV-RL plasmids and incubated with aplykurodin A in the presence or absence of doxor ubicin, an activator of p53 pathway, for 15 hours. Luciferase activities were measured 39 hours after transf ection and reported as relative light unit (RLU) normalized to Renilla luciferase activities. (B) HEK293 cells were co-transfected with NF-kB-FL and pCMV-RL plasmids and incubated with aplykurodin A2 in the pre

sence or absence of aspirin, an activator of NF-kB pathway, for 15 hours. Luciferase activities were measur ed 39 hours after transfection and reported as relative light unit (RLU) normalized to Renilla luciferase act ivities.

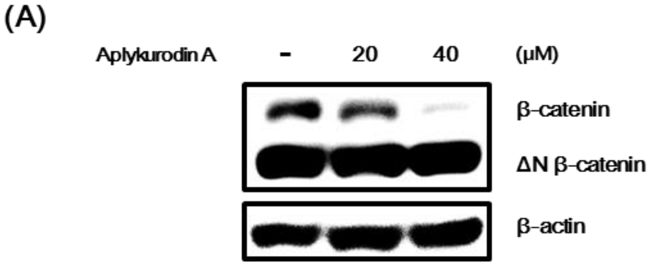

Figure S3. Aplykurodin A promotes β-catenin decomposition in HepG2 HCC cells with a mutant β-catenin lacking the N-terminal phosphorylation motif. (A) The cytoplasmic fractions were isolated and investigated by western blot using anti-β-catenin and anti-β-actin antibodies after each treatment of DMSO (control) an d aplykurodin A (20μM and 40μM) in HepG2 cells for 15 hours.

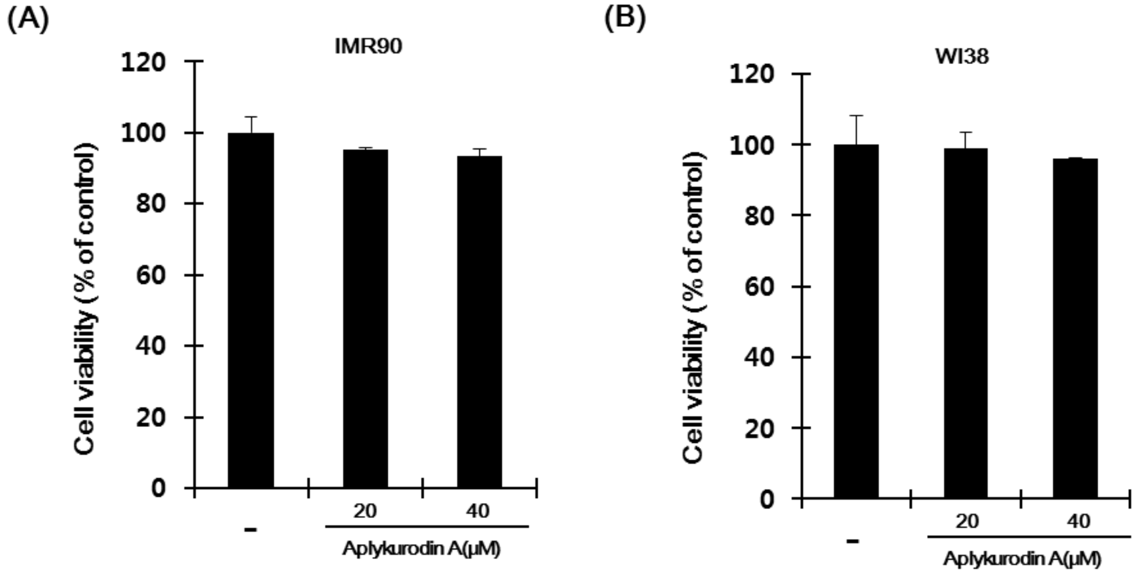

Figure S4. Aplykurodin A is not cytotoxic to IMR90 and WI38 cells. (A) IMR90 cells were incubated with aplykurodin A (20μM and 40μM) for 15 hours and cell viability was measured by Cell titer-Glo. (B) WI38 cells were incubated with aplykurodin A (20μM and 40μM)for 15 hours and cell viability was measured b y Cell titer-Glo.

Table S1. NMR Spectroscopic Data of aplykurodin A (300 MHz, methanol-d_4)

Position	δ_H, mult. (J in Hz)	δ_C
1	—	175.2
2	2.44, m; 2.14, m	38.8
3	2.35, m	34.4
4	3.86, br s	67.4
5	2.14, m; 1.96, m	29.6
6	1.78, m; 1.74, m	30.2
7	—	44.3
8	2.14, m	44.9
9	5.06, d (6.3)	82.5
10	1.93, m; 1.66, m	34.5
11	1.94, m	48.8
12	1.02, s	23.4
13	1.57, m	36.9
14	1.02, d (6.4)	19.2
15	1.46, m; 1.13, m	37.7
16	1.46, m; 1.30, m	25.2
17	1.21, m	40.6
18	1.46, m	29.1
19	0.94, d (6.6)	22.9
20	0.94, d (6.6)	23.2
